# The Baby Box scheme in Scotland: A study of public attitudes and social value

**DOI:** 10.1111/hex.13639

**Published:** 2022-10-28

**Authors:** Zoë Skea, Agata Kostrzewa, Louise Locock, Mairead Black, Heather M. Morgan, Mandy Ryan

**Affiliations:** ^1^ Health Services Research Unit University of Aberdeen Aberdeen UK; ^2^ Obstetrics and Gynaecology, Aberdeen Maternity Hospital, Institute of Applied Health Sciences University of Aberdeen Aberdeen UK; ^3^ Postgraduate Education Group, Institute of Applied Health Sciences University of Aberdeen Aberdeen UK; ^4^ Health Economics Research Unit University of Aberdeen Aberdeen UK

**Keywords:** baby boxes, qualitative health research

## Abstract

**Background:**

The Scottish Government introduced a free Baby Box scheme for all new parents in 2017, modelled on the Finnish scheme, to give every baby ‘an equal start in life’. There is little evidence that it results in better health outcomes, but there has been limited research into different perspectives and discourses on such schemes.

**Methods:**

Four focus groups were conducted with 21 parents in North‐East Scotland. Recordings were transcribed verbatim, anonymized and analysed thematically with NVivo 12 software. Our thematic analysis was both inductive and deductive—remaining open to themes identified by participants themselves but also informed by the social policy literature on universalism and social cohesion.

**Results:**

Across all the focus groups, we found a high degree of positivity about the principle of the Baby Box scheme, and for the most part the practical value of the contents. This was remarkably consistent across different communities and backgrounds. There was little evidence of the strongly polarized views present in media reporting. Parents seemed considerably less focused than the media on safety and health outcomes, and more focused on practical, material and social impacts. They reported little in the way of feeling patronized or monitored by the government.

**Conclusion:**

Our findings have important implications for future economic evaluations of the baby box. Such evaluations should broaden the valuation space beyond health outcomes to allow for the value of feelings of inclusion, solidarity and being part of a community.

**Patient or Public Contribution:**

This small project was designed in response to parent views already collected in the early roll‐out of the Baby Box scheme in Scotland, about their priorities and responses to the scheme. Additional views were sought on the topic guide for the focus groups, and local community groups advised us on recruitment and the best timing and location for the focus groups to be held. The focus groups themselves were conducted as research, but with the intent of ensuring parent views featured more prominently in a debate that has been largely dominated by clinical and public health perspectives.

## 1 INTRODUCTION

The ‘Baby Box’^1^ is a universally available scheme for new parents, introduced by the Scottish Government in August 2017 to ‘give every single baby in Scotland an equal start in life’. It was modelled on a similar scheme that has been running as a universal benefit in Finland since 1949. The Scottish scheme includes a large, decorated cardboard box, which can be used as a crib, and inside the box, a mattress, bedding, new baby clothes, bibs, a towel, toys and baby books, a thermometer and nursing pads. It also contains a poem in the Scots language, ‘Welcome, wee one’. Access to the box is voluntary, although encouraged by health and social care professionals. The Scottish Government reports that, in the scheme's first 3 years of operation boxes were delivered to 144,000 homes, with a 93% take‐up rate.^1^

Much media and research attention has focused on whether the box offers a safe sleeping space, and whether the scheme results in improved health outcomes, such as lower infant mortality and morbidity or higher rates of breastfeeding.^2^ There is little evidence, either from Finland or from Scotland, of improved health outcomes. In Finland, obtaining the box was dependent on registering for state maternity services. The incentive of the baby box, it is argued, encouraged disadvantaged women to receive care, and that this is more likely than the box itself to have reduced infant mortality rates.^3^, ^4^

In a review of the evidence for a number of safe sleep interventions,^5^ it was noted that the provision of cheap portable cribs may help in reducing the risk of co‐sleeping in some communities, but suggest this may be more because of information about safe sleeping and improved parental knowledge than the crib itself.

As well as a lack of evidence for a positive impact on health outcomes, it has been suggested that the boxes may not meet safety and flammability standards, and that the sides may be too high for parents to observe the baby easily.^3^ This contrasts with initiatives in New Zealand to provide a *wahakura* (Māori woven flax basket) or a *pepi‐pod* (plastic box with low, ventilated sides), which are designed to give babies their own sleeping space, but alongside their parents.^6^

There has been little attention in the literature to what parents think of baby boxes and their potential wider social and cultural impacts. In Finland, the box has become a much‐loved cultural tradition, seen as a marker of parenthood and a symbol of equality. Each year new designs are announced and each cohort of babies can be identified by the colour of that year's snowsuit or blanket. Nearly all first‐time parents in Finland take up the offer of a box; a third of parents overall choose a cash payment instead, suggesting many second‐time parents opt for the box again.^7^

Universality and social cohesion may thus be an under‐explored aspect of Baby Box schemes, but universal schemes remain rare. It is often argued that take‐up of means‐tested benefits is low, and that people most in need may be deterred by both the complexity of providing the information needed to make a claim, and the stigma involved in doing so.^8^, ^9^ Means‐testing may look attractive to politicians as a way of targeting resources at those most in need, but may end up as an inefficient way of doing so. While there is some evidence that the inefficiencies of means‐testing have been changing over time, and that policy should focus on making means‐testing work rather than seeking to avoid it, the debate is far from resolved.^10^ In designing this study, we were mindful of this debate.

In particular, we note that Scotland has diverged from England in terms of universalism, for example, making free medicines prescriptions and personal social care universally available.^11^ It is often assumed that universal benefits will be most popular among (and important for) people on lower incomes. However, an analysis of the 2016 European Social Survey, covering 21 countries, reported that attitudes to the idea of completely means‐tested welfare provision were complex.^12^ The analysis found that the upper and middle classes were most opposed to the idea, and that ‘more‐egalitarian people show a higher level of support for means‐testing, even though the political left has traditionally promoted universalism’. Respondents' attitudes were also affected by the national context of different welfare state models, with people living in countries with the less generous provision and higher levels of poverty being more likely to endorse complete means testing. The authors conclude that ‘although it is generally assumed that universal provision is the best strategy to address the needs of disadvantaged people, our results suggest that from an electoral point of view, targeting within universalism may be a more appealing welfare strategy’. Thus what the evidence suggests is most effective in alleviating poverty may conflict with what is electorally most attractive.

A recent study of baby boxes in England notes that most schemes there are a hybrid arrangement between National Health Service providers and commercial companies.^4^ In these schemes, parents sign up to a commercial ‘rewards’ website to claim their box, which mostly contains trial‐size products and discount coupons as well as a fitted mattress and a sheet. The authors note, ‘the intended outcome is to generate profit for the commercial partner, and face‐to‐face engagement for health‐providers to improve child health and well‐being’. However, only 27% of parents surveyed were aware of the commercial nature of the scheme, and few realized they had given contact details to a commercial third party. Parents who took up the offer of a box were generally happy with the ‘free stuff’, though interestingly one parent replied that ‘it seemed a bit sparse in comparison to the Scottish boxes’. Sixty‐eight percent reported they used the box for their baby to sleep for at least some time during the day or night.

In the US, some states are experimenting with baby boxes, but there is little evidence of the experiences of parents who have actually received one. A survey of 541 parents focused on the usefulness, safety and attractiveness of the box as a sleep space, and found little evidence it would be acceptable to parents.^13^ A qualitative study of 28 parents also found ambivalent attitudes; some parents were happy to consider using it as a sleep space, but others were concerned about the stigma of using a box and feeling like their baby was ‘about to be shipped’, even though they thought it might be ‘good for those who can't afford a crib’. Similarly, one person commented that free contents might be helpful for poor families.^14^

The Scottish Baby Box scheme thus remains unusual as a recently introduced universal scheme and offers an opportunity to research how it is received. A small qualitative study commissioned by the Scottish Government from Kantar‐TNS before the introduction of baby boxes found generally positive parental views.^15^ However, the absence of feeding bottles and ‘normal’ (i.e., disposable) nappies was felt to convey a message about socially expected parenting behaviour that not everyone agreed with (i.e., promoting exclusive breastfeeding and reusable nappies).

A pilot evaluation with 34 families was also commissioned from Ipsos‐Mori.^16^ This identified a similarly positive reception overall, but there were mixed views about: the amount of health information to include; uncertainty over the cost‐effectiveness of universal provision; some concern about feeling pressured to breastfeed and dislike of the reusable nappies included. These were removed from subsequent iterations of the box.

Before commencing the fieldwork reported in this paper, we reviewed newspaper reporting in Scotland on the introduction of the baby box (see Supporting Information: Appendix 1). This demonstrated that the voices of parents actually in receipt of boxes were largely missing from the (often highly polarized and politicized) media discourse around baby boxes, which reported mainly the views of politicians and ‘experts’. Safety—or more accurately lack of safety—was a dominant issue in newspaper reporting, with many articles commenting on poor evidence for the safe sleeping and reducing infant mortality (commonly citing the study by Blair et al.^3^), as well as potential fire hazards. One lengthy article in the tabloid The Scottish Sun, which is overtly hostile to the policy of the ruling Scottish National Party (SNP), cited Dr. Blair as claiming the box might be flammable.^17^ Following this, a reporter from the paper attempted to prove how flammable the box was by setting fire to it in his garden, which prompted considerable mockery on Twitter and in the press favourable to the SNP.^18^

Given the absence of parental voices in both the existing literature and media reporting, we conducted focus groups with a range of parents in North‐East Scotland (Aberdeen) who had received or been eligible for the box to assess what mattered to them.

## 2 METHODS

Four focus groups were conducted with 21 participants in total, during February 2020. Participants were recruited using flyers in community centres and university premises and via social media adverts.

The first two focus groups were organized on the University of Aberdeen campus and included university employees (both academic and support staff from a range of grades) and others for whom this was a convenient location, including NHS workers (*n* = 3 and *n* = 7). The other two took place in local community centres: one in the Tillydrone area of Aberdeen, one of the most socially deprived areas of the city (*n* = 4) and one in the affluent neighbourhood of Ferryhill which falls within the least deprived 10% areas in Scotland (*n* = 7).^19^ Local community groups advised us on recruitment and the best timing and location for the focus groups to be held. We also discussed the topic guide with support workers at the two community centres. Focus groups were held either in the evening or during the daytime to accommodate the needs of different groups of parents.

The sample included 18 female and 3 male parents. To minimize perceived barriers to taking part, participants were not required to provide personal details such as occupation or nationality/ethnicity as a condition of taking part. However, many volunteered this information. The majority identified themselves as Scottish, but the sample included several ‘new Scots’, including recent migrants from Poland, Lithuania, Romania, Luxembourg, France and America, and one person of Chinese heritage. Occupations mentioned included NHS workers, university admin and IT staff, students, a social care worker, an oil and gas worker, a nursery manager and a teacher. Some were currently full‐time carers or unemployed.

Each of the four groups lasted between an hour and a half and 2 h, during which time the discussions were audio‐recorded with participants' written consent. Recordings were transcribed verbatim, anonymized and analysed thematically with NVivo 12 software. We conducted a thematic analysis that was both inductive and deductive^20^—remaining open to themes identified by participants themselves but also informed by the social policy literature on universalism and social cohesion. For example, we included codes reflecting prompts around universalism or means‐testing as the basis for making support available, views about the impact on social inequalities/social benefits and also to what extent participants felt the box made them feel supported by the state.

One researcher (A. K.) took primary responsibility for data collection and analysis. Other members of the research team met regularly with A. K.; discussed and refined the coding framework used and supported the development of analytic interpretation.

## 3 RESULTS

Across all the focus groups, we found a high degree of positivity about the principle of the Baby Box scheme, and for the most part the practical value of the contents. This was remarkably consistent across different communities and backgrounds. There was little evidence of the strongly polarized views present in media reporting. Parents seemed considerably less focused than the media on safety and health outcomes, and more focused on practical, material and social impacts. They reported little in the way of feeling patronized or monitored by the government.

Some of the most highly valued aspects of the scheme included the practical support it provides for new parents and the fact that it is available to all parents regardless of their circumstances. Parents described the scheme as being ‘really useful’, ‘exciting’, ‘a lovely gift’ and ‘a good foundation for new parents’:

I was just overwhelmed with how much you actually got in the Baby Box. Like, it's really packed. You can't say that they don't give you enough. There is like, loads in there. The only thing I found funny was the fact that they give you condoms in it <laughs>. I was like ‘they don't want it anymore, they don't want to give you any more boxes <laugh>. (Participant 2, Focus Group 3)

As some parents pointed out, the box contents had things they would not have thought they might need:

I would never think of getting the bath thermometer or even just an ear thermometer, and the fact that you get that in the box is fantastic. (Participant 3, Focus Group 1)

I was really quite excited when it arrived. Like, there were so many nice things in it. Especially for a first time mum, it kind of tells you the sort of things that you need as well, because you are not always sure what you do and don't need. I think we've used most of the stuff, particularly the clothes, the play mat, the duvet, the wrap. Things like that. (Participant 4, Focus Group 3)

Other less positive comments were also expressed and debated, particularly about the box itself as a sleeping space, as well as some of the contents. Some used the box as a sleeping space during the day, or used the mattress but not the box, but commonly people had either bought or been gifted their own Moses basket or crib and preferred those. The box was also regarded as useful for storing toys and other equipment:

…we haven't really used it for sleeping. At the moment it's just storage. (Participant 2, Focus Group 2)

My intention is that when I'm downstairs is that the Baby Box will be used as a sleeping implement, just to save me carting something up and down. Because we did that with the Moses basket and it was just, like, very impractical. So, it is my intention to use it as a daytime sleeping. (Participant 2, Focus Group 1)

In terms of contents, there were some criticisms that the clothes provided weren't the right size; for some with larger birthweight babies 0–3 months sizes were already too small, whereas for others they were too big:

I loved the Baby Box, but I think some of it was quite seasonal, it's not … it might be good, I mean, the jacket, the little grey jacket, I think that is … is it 3 to 6 months I think? But he was born in January, so by the time he was gonna fit that—and it's on a larger side, which is great—but he didn't really get much use out of it, because it wouldn't fit him until summer. (Participant 4, Focus Group 2)

he was born kind of early so again, so again lot of clothes didn't fit and everything just kind of drowned him and … thinking, by the time things do fit him, again, it's not gonna be the right size and what not. For him it's going to be kind of out of season and not appropriate. (Participant 3 Focus Group 2)

Although the various thermometers were seen as useful, there was some concern about the quality of the make and their accuracy:

But there is certain things that we haven't used of it at all, like the thermometers. We found them to be a bit inaccurate. So, like, our house thermometer was showing that it was like 28 degrees in our bedroom, so I was panicking, so I said to the midwife ‘is it 28 degrees in here?’ and I showed it to her and she was like ‘oh no, that's dodgy’ and she said ‘oh no, a lot of them have been faulty’, they've been told. (Participant 4, Focus Group 3)

Safety did not emerge as a major issue, perhaps partly because people were generally not using them as a cot. The Scottish Sun article in which the reporter set fire to a box was used as a prompt during focus groups, and generated considerable scorn and hilarity, but the common view was that the boxes were likely to be safe, even if they chose not to use them much for sleeping.

It is of course likely that people who volunteered to take part already held positive attitudes, and in a focus group setting people may feel constrained to moderate their tone and seek common ground. At the same time, the discussion was thoughtful and reflective. Participants acknowledged legitimate questions of value for money and trade‐offs with other kinds of expenditure, particularly for second and subsequent births, but also acknowledged the possibility of wider effects on social cohesion from making it a universal benefit.

In the following sections, we develop in greater depth the following three themes.

1. Providing an equal start in life.
2. Universal or means‐tested?
3. Additional social benefits of the scheme.

### 3.1 Providing an equal start in life

As noted above, focus group participants reported generally very positive views about the Baby Box scheme, but these were often to do with personal feelings and the wider social ‘message’ of the scheme (see also ‘additional social benefits’ below) rather than whether it would make a big difference to equality. For example, one participant observed that ‘it can make you feel more equal than everybody else, that we are all being treated the same and that somebody is trying to help you’, suggesting the equalizing effect was more a psychosocial sense of being valued and supported as a parent, rather than an equal material start for the baby. One new migrant from America observed:

I thought the stuff in the box was just absolutely lovely and I just thought it was wonderful to think that any child, regardless of their situation would have these lovely, beautiful things that are just for them … So I thought, I love the equaliser idea. And coming from an American family as well, my relatives literally cannot believe that we get this stuff. (Participant 6, Focus Group 2)

Participants were conscious that in material terms the box would not in itself make much difference:

It's definitely a step, but I don't think … it's not enough. if every baby was to get the same start, there would need to be a lot more, if that makes sense. (Participant 2, Focus Group 1)

The high quality of the contents of the clothing and equipment included was widely commended, and prompted one participant to observe how this could affect the perception of visible equality:

That's good, because that means that, you know, by looking at any given child you can't immediately make that judgement about the level of deprivation, or the education of their parents. So it does equalise in that social aspect as well… (Participant 5, Focus Group 2)

### 3.2 Universal or means‐tested?

Closely related to the theme of an equal start in life, the question of whether the scheme should be available universally or only to those who need it most has been the subject of heated debate in Scotland, in the media and in politics. Focus group participants' views tended to be less polarized but echoed similar concerns, within the context of an overall favourable attitude towards the boxes. There were certainly a few who felt that the money could be better spent and that the scheme could prove wasteful:

I know it's funny I'm saying this, because I've had two Baby Boxes, but I'm not sure they should be available to [everyone] … I think it's such a huge thing that everybody gets that you'd think there needs to be some means‐assessment or some sliding scale of what you get … I do wonder if it's sustainable for every child, and whether it's also a little wasteful to give everybody all of this. (Participant 1, Focus Group 4)

One participant suggested the issue of waste could be addressed by offering vouchers to parents who are not interested in receiving another box for a subsequent child, similar to the cash payments offered in Finland.

However, the great majority of participants stated they were in favour of the scheme being available to everyone. Some parents claimed that means‐testing the scheme would ‘take away the main purpose of the box’, and turn the box into a source of social stigma, as this exchange between participants demonstrates:

P3: I think it takes away the purpose of the box. For that equality, that everybody start off with the same thing. If then you start with just a few, you only get it on benefits then it's taking away the kind of main purpose of the box.

P6: And then I think it's stigmatising…

P5: Stigma

P3: You know that the child that's wearing that outfit needed the box rather than it's just something. Because I know, from being in a nursery, a lot of kids come in wearing the grey fleece, hooded jacket and things. Whereas, if you had children coming in it, it causes that kind of a stigma for that child. (Focus Group 2)

One participant compared it to the means‐tested lunch tickets that her partner used to receive as a child:

It always sticks in my mind how my husband was always so embarrassed at school, because he was the only one who had the lunch tickets (…), and he knew that everybody was looking at them when they got the lunch tickets, him and his brother. And I guess it would be kind of like that. You would know that everybody knows that you've got no money, which might not be easy for people, especially with their baby. (Participant 6, Focus Group 2)

In terms of efficiency, it was suggested that means‐testing might increase the administrative cost of the scheme, and result in those most in need either not applying or avoiding using the content to avoid embarrassment. One parent imagined someone thinking to themselves ‘oh, I'm not putting these clothes on today because people will think I've needed a handout’.

Several focus group participants saw universalism through an additional lens of personal guilt about receiving so much support from the state without necessarily needing it. One participant who described the scheme as ‘wonderful’ also commented:

We're given so much, it feels a bit wasteful … I don't know, I feel guilty for it. I feel like I should be somehow giving something back to somebody. It feels like a lot to just be given, there should be a catch somewhere, there should be some sort of, I don't know … reciprocal. (Participant 1, Focus Group 4)

Another mother commented:

I couldn't wait for it to come and when it came, I remember being heavily pregnant and being emotional, like, ‘oh my God, there is so much stuff!’. But I was like, ‘oh, I've not actually, like, paid a penny for this’ and you kind of feel, you feel a little bit greedy about taking it. But at the same time, it's nice to know that actually it's there for every mum and it's there to help you. (Participant 2, Focus Group 3)

Echoing the idea of reciprocity raised above, another participant suggested that receiving the box should be contingent on being willing to contribute to society economically:

You should get [support from the government] if you do want to work, if you do have an income, if you do try to go back to work after maternity leave … there are some people who wouldn't like to go back to work. (Participant 1, Focus Group 1)

### 3.3 Additional social benefits of the scheme

This leads us to our final theme, which builds on the themes of equality and universalism to explore the wider social and psychological impacts of the Baby Box scheme. The idea of social cohesion and identity was a topic of considerable debate in the focus groups.

Although focus group participants generally expressed favourable opinions on the Baby Box scheme and its universal provision, the value attributed to the programme varied significantly between participants. Whilst some parents focused only on the practical benefits of the scheme and struggled to see any connection between the Baby Boxes and wider politics and society in Scotland, others appreciated the symbolic aspects of the scheme as well as the practical ones. Participants from the first focus group in particular (despite being conducted on university premises) expressed a lack of interest in politics, with one interviewee saying that she ‘would just not have really associated the name of the scheme with politics at all’ and another person adding that ‘there are other things to think about than politics when the baby is coming’. Those participants tended to focus on the short‐term, practical benefits of the baby boxes as opposed to the symbolic meaning of it or its potential wider social benefits.

One parent from Focus Group 2, who appear to have more clearly defined political views, referred to the emphasis in Finland on ‘the social support from health visitors and midwives’ as well as offering the maternity package (equivalent of the Baby Box) and argued the Scottish scheme would only make a difference if it followed the same pattern. She explicitly rejected the idea that this might be perceived as a ‘nanny state’:

I think the issue with kind of, the nanny state as a concept is where the state is forcing people to make the choices for the benefit of the state. Where the state is improving access and improving provision and aiming for equality, and then there are sort of opt‐ins and opt‐outs so that people can choose what's suitable for them and their families, then it will probably be derided as being a nanny state because it costs money, but that's actually not what's going on. (Participant 5, Focus Group 2)

There was little support in any of the groups for the idea that the box might constitute government surveillance or interference, and people generally welcomed the inclusion of information leaflets and items to support breastfeeding. One participant from Focus Group 4 who suggested offering vouchers for parents who are not interested in receiving another box for a subsequent child said that the government could monitor what parents are spending these resources on, which would allow the authorities to gain a better understanding of people's needs. When asked whether they would be happy to disclose that information, all participants readily agreed, stressing that people could always opt‐out of such benefits if they did not agree.

For some participants, the message conveyed by the box was crucial. Another male participant from Focus Group 4 stated that to him the symbolic meaning of the Baby Box scheme as a recent migrant was particularly important:

I got a British passport a month ago (…). I'm from Poland, my wife is from Poland. When (…) we got the box, it feels like I belong here. The Scottish government actually gave me something for the child being born here … so it's symbolic more than anything else. (Participant 5, Focus Group 4)

A participant from Focus Group 2 shared a similar view—she stated that it was important to her that other parents who come from different backgrounds are able ‘to identify with me and my family because you know—even if it's just, we had a baby in the same year’ (Participant 5).

Another participant from Focus Group 4 also referred to Scandinavian values and stated that to her the idea of providing a Baby Box to all expecting parents had a symbolic meaning of ‘sharing something’ with other members of society (Participant 3). She perceived it as a good start of a relationship between families and the state: ‘It's kind of society we want to be in, like, be present from the beginning to help you raise your children and just enjoy being parents, so it brings something different that is not just the family or friends, but it's also the whole social thinking’. She mentioned that she was originally from France, and felt ‘very happy I married a Scot’, because if she still lived in France, she would not be able to benefit from useful support for parents such as the Baby Box or extended maternity leave.

This linked to generally very positive wider views on the level of welfare support provided in Scotland and the quality of life in Scotland in general (even though there were some criticisms of service delivery). For instance, as seen below, some participants concluded that the level of welfare support available in Scotland is very high, however, people tend not to notice it:

‘The free prescriptions, the free dental care which you get when you are pregnant, it's fantastic. A lot of these things I think you just take for granted, it's just available to you’. ‘[This discussion] has just kind of brought to the forefront just actually how much the government are actually helping us without you even thinking that they are’. (Participant 3, Focus Group 1)

However, not everyone was equally enthusiastic in their judgements on the quality of life in Scotland. One person referred to continuing social inequalities in the country:

The inequality, that's the real thing we have to address. (…) Scotland is probably a great place to grow up if you have a really good support network behind you, rich affluent parents who know what they are looking for (…) who've got all that sort of social advantages. But not if you're stuck in dodgy housing, overcrowded, or where the services are miles away… (Participant 5, Focus Group 2)

## 4 DISCUSSION

This study demonstrates that the polarized and partisan debate about the Baby Box scheme in Scotland often marginalizes the voices of parents actually in receipt of the box, and does not resonate well with their more positive and practical attitudes. Parents liked most of the contents of the box, and even if they did not always use the box itself for sleeping they found it attractive and often found other uses for it, for example, to store baby equipment or toys. They were variously indifferent to or amused by media alarmism about safety considerations and attempts to demonstrate that the box was a fire hazard, which featured so prominently in media reporting and is also a preoccupation of much of the academic literature.^5^

Our focus group data tends to contradict US research into hypothetical attitudes suggesting that people might feel a degree of stigma, particularly in relation to using the box as a crib.^13^, ^14^ Our participants tended to take a counter view that universal provision would be more likely to avoid stigma and that the feeling of social cohesion among a cohort of parents evident in Finland was also a likely outcome in Scotland.

Participants in the focus groups were generally supportive of universal provision; although they could appreciate arguments against everyone getting the box this tended to be couched more in terms of a personal sense of guilt rather than a belief in targeting and means testing. These findings resonate with the wider long‐running debate in policy studies of means‐testing versus universalism. The Scottish Baby Box is an interesting counter‐example of a newly introduced universal scheme, worthy of further study over time. Our participants' thinking also went beyond the principle of an equal start in life to include a sense of feeling welcomed and cared for as new parents by the state. They rejected the notion that the box exemplified a ‘nanny state’ interfering in their lives.

Of course, those who volunteered for focus groups are likely to have been those who felt positive about it, but nonetheless, this study is an important corrective to the absence of parental voices in other forums and the academic literature and would seem to support the findings of early evaluations commissioned by the Scottish Government.^15^, ^16^ Our sample was located in one geographical area. It is small and pragmatic, with only four participants attending the focus group held in one of the most socially deprived area of the city. It could be argued therefore that most came from backgrounds that possibly did not financially need the box. However, we were reassured that across all four focus groups our participants came from a range of socioeconomic backgrounds and national origins.

Our findings have important implications for future economic evaluations of the baby box. Measures of impact of social and public health interventions generally focus on health outcomes and cost‐effectiveness (e.g., cost per life saved [by e.g., a reduction in sudden infant deaths]) or cost per Quality Adjusted Life Year. Such evaluations fail to capture broader indicators of value identified in our research, including feelings of inclusion, solidarity and being part of a community. Future evaluations should adopt a user‐centred cost–benefit analysis, taking into account all factors important to users in the provision of the baby box. Failure to do this will underestimate the true value to society of the baby box.

## AUTHOR CONTRIBUTIONS

The research team comprised three qualitative social science researchers (Zoë Skea, Louise Locock and Heather M. Morgan), a sociology student Intern (Agata Kostrzewa), a health economist (Mandy Ryan) and an academic obstetrician (Mairead Black). Louise Locock, Mandy Ryan, Zoë Skea, Heather M. Morgan and Mairead Black designed the overall study methods. Agata Kostrzewa conducted all data collection and analyses as a visiting Intern under supervision and input from Zoë Skea, Louise Locock, Mandy Ryan, Heather M. Morgan and Mairead Black. Zoë Skea and Louise Locock wrote the first draft of the manuscript. All authors reviewed and approved the final version.

## CONFLICT OF INTEREST

The authors declare no conflict of interest.

## ETHICS STATEMENT

The study was approved by the Committee for Research Ethics & Governance in Arts, Social Sciences & Business at the University of Aberdeen.

## Supporting information

Supplementary information.

## Data Availability

Research data are not shared. To preserve anonymity as specified under our ethical approval, focus group transcripts are not available for sharing.
