# Supplementary material for: The Baby Box scheme in Scotland: A study of public attitudes and social value
Source: Health Expect. 2022 Oct 28;25(6):3307–14. doi: 10.1111/hex.13639 (PMC9700131; doi:10.1111/hex.13639)
Supplement: Supplementary file 1 — Supplementary information. [file HEX-25--s001.docx]

*Appendix: Preparatory analysis of newspaper articles*

*Methods*

A search was conducted of news articles related to the Scottish baby box scheme in five national Scottish newspapers of diverse political alignments and journalistic style. A key characteristic of Scottish newspapers is the extent to which each aligns or not - or is perceived to - with the campaign for Scottish independence from the rest of the UK, and therefore with the Scottish National Party (SNP), which currently governs as a minority government with support from the Scottish Green Party. We therefore include a brief comment on each newspaper’s stance on the 2014 Scottish independence referendum.

- The Scottish Sun (12 articles) – tabloid, largely conservative stance but has supported SNP, neutral on 2014 referendum
- The Scotsman (27 articles) - broadsheet/compact, no stated political affiliation, backed a ‘no’ vote in 2014
- The Herald (39 articles) – broadsheet, no stated political affiliation, backed a ‘no’ vote in 2014
- The National (26 articles) – compact, aligns with SNP and backed a ‘yes’ vote in 2014
- The Press and Journal (3 articles) – compact, regional paper (covering Aberdeen city and North of Scotland), no stated affiliation, neutral on 2014 referendum

All articles available to August 2019 (the date of the work package) were considered.

Articles were searched using the Lexis Library News database and online editions of each newspaper. Articles were reviewed if they:

1. Included the phrase “baby box” in the headline.
2. Contained at least one paragraph focused primarily on the baby box scheme.

Duplicates and articles not focused primarily on the scheme were excluded. A manual search was also conducted in order to look for other potentially relevant articles which did not have the phrase "baby box" in the headline.

206 articles were retrieved initially. 99 were excluded for not meeting the inclusion criteria, thus the final number analysed was 107.

An initial sentiment analysis of each article was conducted, using the categories: very negative; moderately negative; neutral or mixed opinions; moderately positive; very positive. Article text was also imported into NVivo software, coded and thematically analysed.

*Summary findings*

Figure 1 below shows the spread of views towards baby boxes across all articles, and Figure 2 shows the overall sentiment by newspaper. Reflecting their more avowedly neutral stance, the Herald and the Scotsman show a spread of negative, neutral/mixed and positive pieces. The more conservative-leaning Scottish Sun has more negative than positive reports, while the Press and Journal and the SNP-leaning National have only neutral/mixed or positive pieces.

Figure 1

Figure 2

Safety – or more accurately lack of safety – was a dominant issue, with many articles commenting on poor evidence for the safe sleeping and reducing infant mortality (e.g. Blair et al 2018), as well as potential fire hazards.

Parental voices were almost entirely absent from the articles, with the focus being primarily on political, editorial and scientific commentary. Unsurprisingly, media reporting reflected political cleavages, with a Conservative preference for means-testing and Labour and SNP preferring universalism.

Indirectly representing parental views, *The Herald* quoted Maree Todd, current Children and Early Years Minister: “I think that [universality of the scheme] is one of the nicest things about the box. It’s one of the things that many parents give me very positive feedback about.”

The Scottish Conservatives, conversely, were reported to hold negative opinions on the scheme’s universalism.

Miles Briggs, Scottish Conservative Shadow Cabinet Secretary for Health & Sport, was quoted by *The Scottish Sun* as saying: “We believe limited resources should be focused on the most vulnerable in society, not spread thinly and wasted like we are seeing from the SNP.”

One parental view came from Claire Imrie, a Conservative local councillor from Aberdeen, who was expecting a baby in August 2018. The Press and Journal reported that Ms Imrie stated that the SNP’s initiatives, such as the baby boxes, were *‘unnecessary provisions’* which should only be given to those *‘in most need’*.

By contrast, The National quoted Gayle Mellor, a mum of three from Alloa, as saying: “[The box is] brilliant – a lot bigger than I thought it would be….It’s giving all babies the same start in life which I think is the main reason for the launch, and that’s a good thing.”
